# Self-Diagnosis of Malaria by Travelers and Expatriates: Assessment of Malaria Rapid Diagnostic Tests Available on the Internet

**DOI:** 10.1371/journal.pone.0053102

**Published:** 2013-01-02

**Authors:** Jessica Maltha, Philippe Gillet, Marloes Heutmekers, Emmanuel Bottieau, Alfons Van Gompel, Jan Jacobs

**Affiliations:** 1 Department of Clinical Sciences, Institute of Tropical Medicine, Antwerp, Belgium; 2 Center for Molecular and Vascular Biology, University of Leuven, Leuven, Belgium; Centro de Pesquisa Rene Rachou/Fundação Oswaldo Cruz (Fiocruz-Minas), Brazil

## Abstract

**Introduction:**

In the past malaria rapid diagnostic tests (RDTs) for self-diagnosis by travelers were considered suboptimal due to poor performance. Nowadays RDTs for self-diagnosis are marketed and available through the internet. The present study assessed RDT products marketed for self-diagnosis for diagnostic accuracy and quality of labeling, content and instructions for use (IFU).

**Methods:**

Diagnostic accuracy of eight RDT products was assessed with a panel of stored whole blood samples comprising the four *Plasmodium* species (n = 90) as well as *Plasmodium* negative samples (n = 10). IFUs were assessed for quality of description of procedure and interpretation and for lay-out and readability level. Errors in packaging and content were recorded.

**Results:**

Two products gave false-positive test lines in 70% and 80% of *Plasmodium* negative samples, precluding their use. Of the remaining products, 4/6 had good to excellent sensitivity for the diagnosis of *Plasmodium falciparum* (98.2%–100.0%) and *Plasmodium vivax* (93.3%–100.0%). Sensitivity for *Plasmodium ovale* and *Plasmodium malariae* diagnosis was poor (6.7%–80.0%). All but one product yielded false-positive test lines after reading beyond the recommended reading time. Problems with labeling (not specifying target antigens (n = 3), and content (desiccant with no humidity indicator (n = 6)) were observed. IFUs had major shortcomings in description of test procedure and interpretation, poor readability and lay-out and user-unfriendly typography. Strategic issues (*e.g.* the need for repeat testing and reasons for false-negative tests) were not addressed in any of the IFUs.

**Conclusion:**

Diagnostic accuracy of RDTs for self-diagnosis was variable, with only 4/8 RDT products being reliable for the diagnosis of *P. falciparum* and *P. vivax,* and none for *P. ovale* and *P. malariae*. RDTs for self-diagnosis need improvements in IFUs (content and user-friendliness), labeling and content before they can be considered for self-diagnosis by the traveler.

## Introduction

In the nineties, malaria rapid diagnostic tests (RDTs) were suggested for self-diagnosis by travelers [1]. The idea was however abandoned after several studies had shown poor test performance and difficulties in interpretation of test results by the ill traveler [1]–[4]. Since then, major progresses have been made in RDT design and performance. Most of the currently marketed RDTs are so-called one-step RDTs that are more simple and user-friendly [5] compared to the multi-step RDT products evaluated in previous studies. Malaria RDTs are now easy to use handheld cassettes detecting antigens produced by the *Plasmodium* parasite which become visible as colored (mostly red) test lines within 20 minutes [6].

Recently, the decline in malaria burden in many parts of the world has made stand-by emergency treatment (SBET) more attractive for many travelers than the classic chemoprophylaxis. In the SBET strategy, travelers and expatriates to low-resource endemic settings carry an emergency malaria treatment (with reliable activity against *P. falciparum*) for self-administration when no medical attention is rapidly available. This option may be considered where the risk of adverse reaction to malaria chemoprophylaxis outweighs the risk of malaria infection [7], [8] and is increasingly promoted by some experts in travel medicine [9]. Self-diagnosis of febrile illness with reliable malaria RDTs could accelerate early therapy (with the standby treatment), preventing complications and death, or avoid unnecessary use of antimalarials [10], [11].

Nowadays, RDTs for malaria self-diagnosis are available through the internet, but their diagnostic accuracy and ease of use have not yet been studied. Also, the quality of instructions for use (IFU) – which assure correct performance and interpretation of the RDT results [4] - can vary widely [12]. Therefore, the present study assessed both diagnostic accuracy and quality of packaging, labeling and IFU of RDT products marketed for self-diagnosis by travelers.

## Methods

### Ethics Statement

The study was approved by the Institutional Review Board of ITM and by the Ethical Committee of Antwerp University, Belgium. RDTs were performed on stored blood samples (“leftovers”) obtained as part of routine diagnostic work-up in international travelers suspected of malaria. In view of the absence of risk and the anonymous data processing, IRB deemed informed consent obsolete.

### Study Design

RDT products for self-diagnosis were evaluated for diagnostic accuracy against a panel of 100 stored blood samples obtained in travelers suspected of malaria and for the quality of packaging and their IFU.

### Patients and Samples

A total of 100 stored EDTA blood samples (including the four human *Plasmodium* species as well as malaria negative samples, Table 1) obtained from returned international travelers clinically suspected of malaria were selected. Samples were collected between October 2007 and September 2011 and were stored at −70°C at the Institute of Tropical Medicine (ITM), Antwerp, Belgium. Species identification was done by expert microscopy, corrected by four-primer real-time PCR [13].

**Table 1:** Panel of clinical samples used to assess the test characteristics of the RDT products.

		Region of travel			
Species	Parasite density/µl median (range)	Africa	Asia	Caribbean/South-America	No data
*P. falciparum* (n = 55)	2,928 (21–1,750,000)	51		3	1
*P. vivax* (n = 15)	1,068.5 (15–14,228)	6	6	1	2
*P. ovale* (n = 15)	817.5 (51–5,930)	14			1
*P. malariae* (n = 5)	382 (26–1,920)	5			1
Negative for malaria (n = 10)	–	8	1		1

### Malaria Rapid Diagnostic Tests

Availability of RDT products for self-diagnosis on the internet was assessed using the search engine ‘Google’. The following terms were used in both English and French: ‘Malaria self-diagnosis’, ‘Malaria self-test’, ‘Malaria survival kit’, ‘Malaria home testing’, ‘Malaria autotest’. Three times a search was performed (January, April and June 2011). In addition, two manufacturers were contacted directly for availability of RDT products marketed for self-diagnosis (Standard Diagnostics and Access Bio Inc.). Another manufacturer (TODA PHARMA) had contacted himself ITM to inform that he had RDTs available for self-diagnosis.

### Test Procedures

Tests were performed according to the manufacturer’s instructions, except that a transfer pipette (Finnpipette, Helsinki, Finland) was used instead of the transfer device supplied with the RDT products. The first observer was the one who performed the test and read test results within the specified reading time. The second observer read test results within 5 additional minutes and was blinded to the results of the first observer. Both observers were blinded to the results of microscopy and PCR. The results of the first observer were considered and compared to the second observer to determine interobserver agreement. Test lines were scored for line intensity as described previously [14]. Faint intensity implies a barely visible test line which risks being interpreted as negative. In case of absence of the control line, the result was invalid and considered negative because it was assumed that travelers will not always have a second RDT available. In order to assess false positive results occurring upon reading beyond the recommended reading time (so-called “back-flow phenomenon” [15], the first observer scored test lines again at the end of the day, between 2 and 8 hours after initial reading.

### RDT Packaging and Instructions for Use

RDTs’ packaging and instructions for use (IFU) were assessed using a checklist adapted from a previous study [12]. In addition, typography, lay-out and readability level of the IFUs was assessed as previously described [12]. Font size was measured in Cicero as the ‘kp’ distance. For health instructions in general, font sizes >12 are recommended, interline spacing ≥2 and fonts of open letter types [16]. The readability level was expressed as Flesh Kincaid grade level. There are no criteria of readability levels for IFUs but for patient education files, a level ≤6^th^ grade is recommended [16].

Some RDT products (OptiMAL and TODA) were delivered as boxes containing multiple single-use RDT packages with IFUs supplied in the boxes as well as in the individual packages. In these cases only the IFUs included in the individual packages were considered, as this IFU will most likely be the one available to the traveler.

### Statistical Analysis

For *P. falciparum* diagnosis, sensitivity was defined as follows: the number of *P. falciparum* samples with a visible *P. falciparum* specific test line, divided by the total number of *P. falciparum* samples. For the non-*falciparum* species, sensitivity was defined as the number of non-*falciparum* samples with a visible pan-pLDH test line, divided by the total number of non-*falciparum* samples. As one product (OneStep, Table 2) detects *P. vivax* instead of all non-*falciparum* species, sensitivity was defined as all *P. vivax* samples with a visible *P. vivax* test line divided by the total number of *P. vivax* samples. Sensitivity was calculated with 95% confidence intervals (C.I.).

**Table 2:** Overview of RDT products and their lot numbers.

Product name	Manufacturer	Further referred to as	Target antigens	Lot numbers	CE label	Recommended storage temperature	Numberof testsper kit	Price pertest (€)	Link to theproduct*
Carestart™ Malaria HRP2/pLDH(Pf/PAN) COMBO Test	Access Bio, Inc., New Jersey,USA	CareStart	PfHRP2/pan-pLDH	AIIIR F40IR	Yes	4–30°C	5	7	
Malaria Curative Kit, Immunoquick	BioSynex, Strasbourg France	Immunoquick	Pf†	SMD031411 SMD081611	Yes	4–30°C	3	21	http://www.smihealth.com/wp-content/uploads/2010/03/HKP001E2.pdf
Labstix Malaria Travel Kit	Labstix Diagnostics (Pty) Ltd., Faerie Glen, South-Africa	Labstix	PfHRP2/pan-pLDH	F1122G1B	No	No temperature displayed	3	6	http://labstix.co.za/products-page/infectious-diseases-other/labstix-malaria-travel-kit/
One Step Malaria (P.F/P.V) Test	Not displayed	OneStep	Pf/Pv†	2010070906	No	4–30°C	1	18	www.std-home-test.com/malaria.html
OptiMAL-IT	Bio-Rad, Marnes-la-Coquette, France	OptiMAL	Pf-pLDH/pan-pLDH	OAOO35M	Yes	2–30°C	24	2.5	www.tcsbiosciences.co.uk/optimal_it.php
Malaria Combo Test	Sanitoets Closed Corporation, Pretoria, South-Africa	Sanitoets	PfHRP2/pan-pLDH	50034 5000	No‡	No temperature displayed	2	12.5	www.anytestkits.com/malaria-test-kit.htm#Buying from us
SD BIOLINE Malaria Ag P.f/PanPOCT	Standard Diagnostics, Hagal-dong, Korea	SDFK63	PfHRP2/pan†	90122	Yes	1–40°C	1	4	
TODA MALARIADIAG 4+	TODA PHARMA, Brussels,Belgium	TODA	PfHRP2/pan-pLDH	K10M4C030111B-1	Yes	2–30°C	1	10	

PfHRP2 =  *P. falciparum* Histidine-rich protein-2; pan-pLDH = pan *Plasmodium* lactate dehydrogenase; Pf-pLDH = *P. falciparum*-pLDH.

* Websites last accessed on 07-08-2012.

† Target antigens not specified.

‡ On the website a CE label was displayed but not on the delivered products.

False positive test lines were defined as any visible test line among the *Plasmodium* negative samples, non-*falciparum* samples generating a visible *P. falciparum*-specific test line and in addition for OneStep a visible *P. vivax* test line generated by *P. falciparum*, *P. ovale* or *P. malariae* samples.

Inter-observer agreement for positive and negative readings and line intensity was expressed by kappa values (κ). In line with previous RDT evaluations κ 0.60–0.80 was considered good and κ >0.80 excellent.

### Blood Lancets and Transfer Devices

The type of blood lancet (*e.g.* safety lancet with retractable needle, safety seal lancet, simple lancet) and transfer device (pipette, straw, loop, inverted cup) was recorded. Besides, blood lancets were assessed for possibility of reuse. The safety lancets with retractable needle consist of a plastic casing in which a needle is fixed. The system has to be primed and next the needle is launched by pushing on a button. For these systems it was checked that the needle could not be launched inadvertently (*e.g.* during transport in the luggage of the traveler) before the protective cap was removed.

### Additional Analysis

To assess the presence of histidine-rich protein-2 (PfHRP2) in one *P. vivax* sample showing a *P. falciparum* test line of strong and weak intensity in four and one out of five PfHRP2-detecting RDTs, a PfHRP2 ELISA (Standard Diagnostics, Hagal-Dong, Korea) was performed.

## Results

### RDT Selection

**Figure 1:**
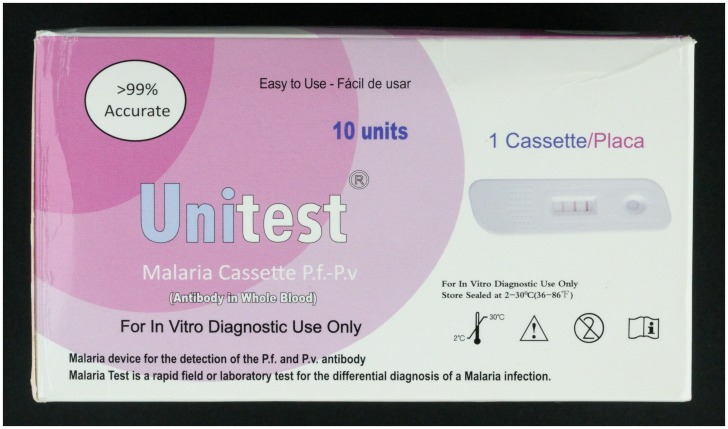
Received product of Unitest (Ciriano global S.L., Zaragoza, Spain). The delivered Unitest malaria cassette P.f-P.v kit mentions detection of antibodies while the almost identical kit on the website mentions detection of the Pf and Pv antigen. (http://www.clinica.co.za/index.php?page=shop.product_details&flypage=flypage.tpl&product_id=11&category_id=3&option=com_virtuemart&Itemid=90).

During the internet search eight RDT products were encountered and ordered. Three of these products were not included in the final selection: one (Malapack Travel test, http://www.vaccinations.com.au/product.htm) was not marketed anymore, the manufacturer of the second product (EZ-Trust Malaria Rapid Screen Test Kit, CS Innovation Pte Ltd, Singapore) replied not to start up the production for an order less than 10,000 tests. The third product that was delivered (Unitest malaria cassette Pf-Pv, Ciriano global S.L., Zaragoza, Spain) appeared to be an RDT detecting malaria antibodies instead of antigens (Figure 1), whereas the product ordered from the manufacturer’s website clearly mentioned an antigen-detecting product.

In addition to the five ordered RDT products, the three tests directly provided by the manufacturers (as explained above) were also included, so that a total of eight RDT products was evaluated (Table 2). Six RDT formats were cassettes, there was one hybrid format (OptiMAL) and one RDT (Immunoquick) consisted of a dipstick.

### RDT Performance

The number of samples detected and the number of false positive lines for malaria negative samples for each RDT product are displayed in Table 3. For 6/8 RDT products 100% sensitivity was reached for *P. falciparum* diagnosis, but Labstix and Onestep showed false positive lines in respectively 80% and 70% of malaria negative samples. Three RDT products detected all *P. vivax* samples. *P. ovale* and *P. malariae* detection was in general poor, Labstix was the only one diagnosing all samples but this was at the expense of poor specificity as this product generated visible pan-pLDH lines in 8/10 *Plasmodium* negative samples as well.

**Table 3:** Test characteristics of the different RDT products.

	Number of samples identified (%)					False positive lines		
	*P. falciparum*		*P. vivax*	*P. ovale*	*P. malariae*	Pv, Po and Pm (n = 35)	malaria negative (n = 10)	
RDT product	PD <1,000/µl(n = 15)	PD >1,000/µl (n = 40)	(n = 15)	(n = 15)	(n = 5)	Pf test line	Pf test line	Pan/Pv test line
CareStart	15 (100)	40 (100)	15 (100)	4 (26.7)	3 (60.0)	4 (11.4)*		
Immunoquick	15 (100)	40 (100)				4 (11.4)		
Labstix	15 (100)	40 (100)	15 (100)	15 (100)	15 (100)	33 (94.3)*	8 (80.0)	8 (80.0)
OneStep	15 (100)	40 (100)	12 (80.0)			28 (80.0)	7 (70.0)	3 (30.0)
OptiMAL	11 (73.3)	39 (97.5)†	13 (86.7)‡	1 (6.7)	3 (60.0)	2 (5.7)	1 (10.0)	1 (10.0)
Sanitoets	13 (86.7)	39 (97.5)†	12 (80.0)^§^	7 (46.7)	2 (40.0)	2 (5.7)*		
SDFK63	15 (100)	40 (100)	15 (100)	1 (6.7)	3 (60.0)	3 (8.6)*		
TODA	15 (100)	40 (100)	14 (93.3)	3 (20.0)	4 (80.0)	1 (2.9)*		1 (10.0)

PD = parasite density, Pf = *P. falciparum*, Pv = *P. vivax*, Po = *P. ovale*, Pm = *P. malariae.*

* including one P. vivax sample that generated a strong positive result upon testing PfHRP2 ELISA.

† one sample missed with parasite density 2,458/µl.

‡ including one invalid result. § including one missed sample with parasite density 3,251/µl.

Cross-reactions of non-*falciparum* samples with the *P. falciparum* test line occurred for the majority of samples in Labstix (94.3%) and OneStep (80.0%). In addition for OneStep 47 *P. falciparum* samples and 12 P. *ovale/P. malariae* samples showed a visible *P. vivax* test line.

### Faint Test Lines

For *P. falciparum* diagnosis, the median percentage of correctly identified test lines with faint line intensity per RDT product was 3.6% (range 0.0%–14.0%). For the correctly identified non-*falciparum* samples, faint line intensities occurred at a median frequency of 23.8% (range 10%–58.3%).

### Interobserver Agreement

Median κ for positive/negative readings for the *P. falciparum* test line was 0.95 (range 0.50–1.00, OneStep κ = 0.50), for the non-*falciparum* test line this was 0.91 (range 0.66–1.00). For line intensity readings median κ were 0.83 (range 0.70–0.96) and 0.76 (range 0.71–0.85) for the *P. falciparum* and non-*falciparum* lines respectively.

### Reading Beyond the Recommended Reading Time

For all but one (SDFK63) products false positive test lines were seen after reading beyond the recommended reading time among 10.0%–90.0% of the malaria negative samples and 9.4%–100% of the non-*falciparum* samples with initially no false positive test lines. Immunoquick, TODA and Labstix were mainly affected with nearly half (>44.4%) of malaria negative samples erroneously diagnosed as malaria and the majority (>88.2%) of non-*falciparum* samples diagnosed as *P. falciparum* malaria upon reading beyond the recommended time.

### RDT Packaging, Content and Design

Half of RDT products assessed had inconsistencies in their names displayed on the outer packaging, the device packaging and the IFU. Moreover, 3/8 RDT products did not mention their target antigens and 2/8 RDT products did not display recommended storage temperature (Table 2). In general, the RDT products contained all material needed to perform the test, however to open the buffer vial of Labstix, a scissor was needed but this was not mentioned among the required materials, only in the procedural steps of the IFU. Only 2/8 (25.0%) RDT products contained a correct desiccant – *i.e.* provided with a color-based humidity indicator allowing to control for humidity saturation. Two products did not contain a transfer device, the drop of blood had to be applied directly to the test strip. The pipettes supplied with Labstix and OneStep did not contain a mark for indication of the correct volume of blood.

For 4/7 RDT devices test line labeling consisted of acronyms (‘Pf’, ‘P’, ‘Pan’), the others used either letters (T) or numbers. Immunoquick contained no labeling at all as it is a dipstick without cassette. Incorrect labeling of the reading window was found for Sanitoets: only the symbols ‘C’ and ‘T’ were displayed at either side of the reading window while the strip contains a control line (“C”) and two test lines. For Labstix discordances were observed between labeling on the test device (Pf, Pan) and the pictures displayed in the IFU (T2, T1).

### Instructions for Use

OneStep did not deliver an IFU with the tests, nor a link to the online version. On the website where the product was ordered an IFU was found. The IFU of OptiMAL consisted only of pictures. None of the IFUs fulfilled requirements for correct font size (>12), median font size was 5.0 (range 4.5–9). Only two products used an interline distance of 2 and six IFUs used an open character. Median Flesh Kincaid grade level was 8.32 (5.86–9.65), only Immunoquick had a Flesh Kincaid grade level ≤6^th^ grade.

Shortcomings of the IFU with regard to test procedure and interpretation are displayed in Table 4. Several critical procedural steps were missing in more than half of the IFUs. For three RDT products, the correct use of the blood lancet was not clearly described or depicted. Sanitoets depicted another type of pipette in the IFU than was delivered in the kit. Moreover, Sanitoets mentioned to cut the end of the sealed pipette that contained the reagent while in the kit a buffer vial with a screw cap was included. Four IFUs failed to mention that the test should not be read beyond the recommended reading time and one IFU (TODA) did not display a reading time at all (for this product, information about reading time was withheld from the box that contained the single packages used for self-diagnosis).

**Table 4:** Presence of important items that need to be addressed in the instructions for use.

Procedure section	Care-Start	Immuno-quick	Labstix	OneStep	OptiMAL	Sani-toets	SDFK63	TODA
Do not use the RDT if the device package is damaged	No	Yes	No	Yes	No	No	Yes	No
Do not use beyond the expiry date	Yes	No	No	Yes	No	No	Yes	Yes
Use the device immediately after opening	No	No	No	No	No	Yes	Yes	No
Place the device on a level surface	No	N.A	No	Yes	N.A.	Yes	No	Yes
Check the desiccant for signs of exposure to humidity	No	No	No	No	No	No	No	No
Disinfect finger with alcohol wipe	Yes	Yes	Yes	Yes	No	Yes	Yes	Yes
Allow the finger to dry before pricking	No	No	No	Yes	No	Yes	No	Yes
Correct use of blood lancet is clearly described/depicted	No	Yes	Yes	Yes	No	Yes	No	Yes
The volume of blood to be transferred is clearly mentioned	Yes	Yes	Yes	No	Yes	No	Yes	No
Hold the buffer vial vertically	No	No	No	No	No	No	Yes	No
Do not use another buffer than the one provided in the kit	No	No	No	No	No	No	No	No
Use an adequate light source for reading	No	No	No	No	No	No	No	No
Do not read beyond the recommended reading time	No	Yes	Yes	Yes	No	Yes	No	No
Interpretation section								
All possible line combinations for invalid test results	No	No	Yes	Yes	No	Yes	Yes	No
All possible test line combinations for positive test results	Yes	Yes	Yes	Yes	No	Yes	Yes	No
Interpretation of a faint test line as positive	No	No	Yes	No	No	No	No	No
Causes of false negative results, in particular low parasitedensities	No	No	No	No	No	No	No	No
Causes of false positive results, *e.g.* presence of the rheumatoidfactor	No	No	No	No	No	No	No	No
A negative test does not rule out malaria	No	No	No	No	No	Yes	No	No
In case of a negative RDT result and persistent suspicion ofmalaria repeat the test after 6–12 h or go to a doctor	No	No* †	No*	No	No	No	No	No
Do not use the test to follow-up treatment	No	No	No	No	No	No	No	No‡
In case of a positive RDT result consult a doctor	No	No*	No*	No	No	Yes	No	No

N.A. = not applicable.

* The user is advised to take the treatment included in the kit.

† Repeating the test after 12 hours is advised, independent of persistence of symptoms.

‡ The user is advised to use the ‘pan’ line for treatment follow-up.

OneStep did not contain any pictures of possible test results in the interpretation section. OptiMAL and TODA did not mention a visible control line and single visible *P. falciparum* test line as a possible result. Following their instructions, a *P. falciparum* infection always generates both a visible *P. falciparum* and pan-pLDH test line. In the present study however, OptiMAL and TODA showed a single visible *P. falciparum* test line among one and three *P. falciparum* samples respectively, leading to 49/55 (89.0%) and 52/55 (94.5%) correctly identified *P. falciparum* samples respectively. IFUs that did not display all possible combinations for invalid results generally only depicted a test in which none of the lines were visible. For TODA, an error was observed on the figure showing the invalid results, *i.e.* a cassette was displayed with a visible control line and an absent test line. Except for Labstix that depicted a less visible test lines among the positive results, none of the RDT products mentioned to consider a faint test line as a positive one. Likewise, causes of false negative and false positive results were not mentioned. SDFK63 was the single product mentioning that a negative test does not rule out malaria. Hardly any advices about test policy were made, except for TODA mentioning to use the ‘pan’ line for treatment follow-up of a *P. falciparum* infection and Labstix and Immunoquick advised to start treatment in case of a positive test or in case of a negative test and persistence of symptoms (Table 4).

### Blood Lancets and Transfer Devices

**Figure 2:**
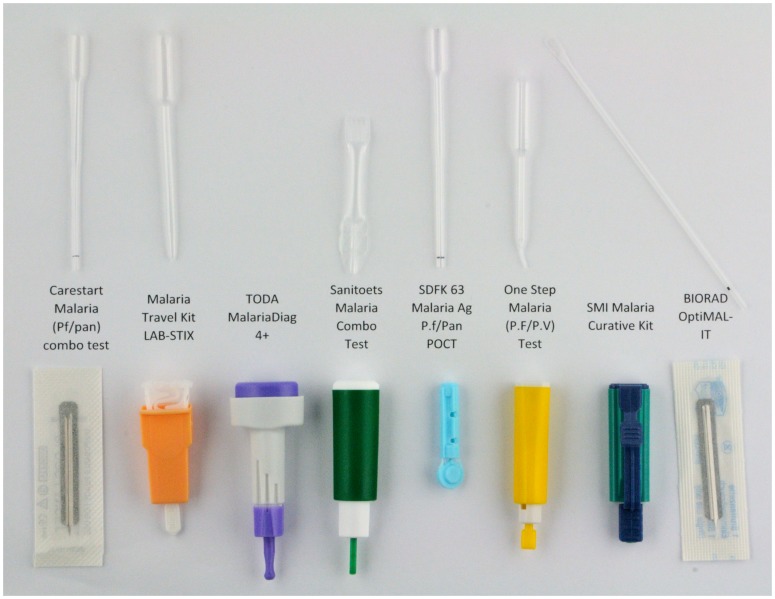
Lancets and transfer devices delivered with the different RDT products. CareStart and OptiMAL included a simple lancet, SDFK63 a safety seal lancet and the other products safety lancets with a retractable needle. The systems of TODA and Immunoquick (SMI) do not require a transfer device (direct contact of the test strip with the drop of blood). Sanitoets contained a calibrated pipette and OptiMAL a straw pipette. The other products included a balloon pipette. The transfer devices of OneStep and Labstix did not display a volume mark.

Blood lancets and transfer devices for each RDT product are depicted in Figure 2. Five lancets were so-called safety lancets with a retractable needle. For two of them reuse was possible and for the other three the needle could inadvertently be launched before removal of the protective cap. Two products did not contain a transfer device, the drop of blood had to be applied directly to the test strip. The pipettes supplied with Labstix and OneStep did not contain a mark for indication of the correct volume of blood.

## Discussion

The present study assessed the diagnostic accuracy of malaria RDT products available for self-diagnosis as well as the quality of packaging and content, the readability level and lay-out of its IFU. Sensitivity was variable and for two products an unacceptable high number of false positive test lines occurred. All but one product yielded false-positive test lines upon reading beyond the recommended reading time. Major shortcomings in IFU were observed among all RDT products and the IFUs were not user-friendly.

### Limitations and Strengths

The present study used a limited and selected number of samples, precluding calculation of predictive values and providing wide confidence intervals for non-falciparum results. Furthermore, RDTs were performed by trained personnel which allowed calculation of RDT sensitivity under perfect conditions but the study design did not include actual performance by the intended end-user. Likewise, ease of use and potential errors of RDT performance as well as interpretation by travelers were not assessed. However, the present study used a collection of samples comprising all four human *Plasmodium* species at different parasite densities providing relevant data on diagnostic accuracy. Besides, the systematic evaluation of RDT packaging and its IFU allowed direct comparison between products and assessment of major shortcomings.

### Accuracy for the Diagnosis of Malaria

*P. falciparum* is the most dangerous species and accurate diagnosis should be guaranteed, even at low parasite densities since the non-immune traveler may already have symptoms at parasite densities of 100/µl [17]. Six RDT products had a good sensitivity for *P. falciparum* diagnosis. The use of Labstix and OneStep is however precluded due to the unacceptable high number of false positive test lines. Among the remaining four RDT products, Immunoquick is less suitable as it only detects *P. falciparum* and in most low endemic areas where SBET is considered *P. vivax* is prevalent as well. SDFK63, CareStart and TODA had good sensitivities for both *P. falciparum* and *P. vivax* diagnosis, but detection of *P. ovale* and *P. malariae* was poor, which is a known phenomenon among RDTs [14], [18]–[21]. IFUs should mention these limitations, and advise to repeat a negative test after several hours or to seek reliable health care if symptoms persist.

The high number of false positive test lines for Labstix and OneStep may be due to problems of non-specific binding including buffer composition [22] and a redesign is needed. Moreover, the high number of false positive *P. vivax* test lines for OneStep may be due to wrong citing of the target antigen which presumably detects pan-pLDH rather than P. vivax-pLDH, which has been described previously for other products [23]. The other false positive test lines occurred at random and may be due to non-specific reactions [24].

The *P. vivax* sample showing clearly visible test lines in all PfHRP2-detecting RDT products was probably obtained from a patient with a recent *P. falciparum* infection and PfHRP2 persistence [25] as the presence of PfHRP2 in the blood was confirmed by ELISA.

Faint test line intensity of true positive test lines occurred mainly among the non-*falciparum* species, although for OptiMAL >10% of *P. falciparum* samples also generated a faint *P. falciparum* test line. Faint test lines are of concern as they tend to be regarded as negative [26] and will not be visible under unfavorable light conditions.

It is possible that the ill traveler will check his RDT again after a few hours to make sure it was really negative. After reading beyond the recommended reading time, false positive test lines may occur as demonstrated in the present study, due to the back-flow phenomenon [15]. Therefore, it is important that the IFU clearly mentions that reading test results should be performed within the time specified in the IFU and any test line becoming visible beyond the recommended reading time should be ignored. For OneStep even reading a few minutes too late resulted in some false positive *P. falciparum* test lines observed by the second observer and explaining the low interobserver agreement.

### RDT Shortcomings in Labeling

The most serious encountered error in labeling was that on the website of UNITEST malaria antigen detection was clearly mentioned and displayed on the picture, while the product delivered targeted malaria antibodies. Apart from errors in the online ordering system, it is of note that both products are nearly identical except for differences in the text present on the box (Figure 1).

### RDT Problems in Design

Some observed errors in design may affect performance under field conditions like errors in labeling of the reading frame and the need of scissors for opening the buffer vial while not mentioned on the package or in the IFU. The lack of a mark on the transfer device to indicate the correct blood volume risks the application of too much or too little blood, leading to poor background clearance obscuring the test lines or to false negative results respectively. The system of direct application of the cassette on the drop of blood looks attractive, but there are no published data about its correctness and ease of use. The most accurate, easy to use and preferred transfer device by health workers in malaria endemic setting was the inverted cup [27] and it can be assumed that this will apply to travelers too.

### Instructions for Use: Procedure and Interpretation

The presently found shortcomings in the IFU are of concern as past evaluation showed problems in RDT performance by the ill traveler which improved after revision of the IFU [4]. None of the IFUs was written to the level of the end-user (high readability level, poor lay-out and user unfriendly typography), which is a crucial requirement for products intended for self-diagnosis [28].

Many of the shortcomings mentioned in Table 4 apply to the use of RDTs by any end-user and have been observed in products intended for laboratory use before [12]. Of particular interest to the layman traveler are to check integrity and expiry date of the product as it can be assumed that – despite the long shelf-life of RDTs, storage periods can exceed those indicated by the expiry dates. Moreover, the traveler, and especially the backpacker, will not always be able to adhere to storage conditions (for most RDT products below 30°C) leading to RDT degradation. Further, the curious traveler might open the RDT packaging before intended use, particularly when the IFU is included in a single pouch together with the device, and by doing so he will expose the product to humidity degradation.

The failure to explain/depict the use of the blood lancet is a major shortcoming as one of the major difficulties in RDT performance by travelers observed in previous studies was the finger prick [2], [29]. Test interpretation was another frequently observed difficulty [2], [3] and therefore all possible test results should preferably be depicted. Of note, information regarding RDT strategic issues (repeat testing, reasons for false negative tests) was poor and when available not always correct *i.e.* using the pan-pLDH line for treatment follow-up, which is debatable, as also gametocytes produce pLDH [30], [31].

### What can be Done to Improve RDTs for Self-diagnosis?

First of all, an accurate performance needs to be assured. Products like Labstix and OneStep performed insufficiently, they contained no CE mark but were actually delivered to users in the European Union. Next, IFUs should give understandable information about the product performance, including the limitations for the diagnosis of the non-*falciparum* species. Furthermore, the IFU needs to become more user-friendly and the procedure and interpretation sections need to be completed at least with the topics mentioned in Table 4. Also for a traveler, multiple lancets, transfer devices and alcohol wipes are advised. A tag for temperature control (*i.e.* a small device or sticker that changes color when the maximal temperature has been exceeded) may be of additional value. Important, fulfillment of all these requirements does not preclude the need for training and counseling of the end-user of these tests. Although not presently studied, previous reports have demonstrated the needs for training and the benefits of a comprehensive training program [29]. For expatriates and travelers performing RDTs abroad who ask for advice, we currently ask them to send a photograph of the RDT. Future technical developments, such as cell-phone based RDT readers should be assessed for applications [32].

### Conclusion

Diagnostic accuracy of currently on the internet available RDTs for self-diagnosis is variable. Based on the present study, 3/8 RDT products are reliable for *P. falciparum* and *P. vivax* diagnosis and one for *P. falciparum* diagnosis only. Instructions on test performance, interpretation and limitations and what to do with test results were incomplete and unsatisfactory for all RDT products. The presently observed shortcomings need to be urgently adapted before RDTs can indeed be used for self-diagnosis by the traveler and expatriate.
